# Validation of SocialBit as a smartwatch algorithm for social interaction detection in a clinical population

**DOI:** 10.1038/s41598-026-37746-x

**Published:** 2026-02-04

**Authors:** Amar Dhand, Samuel Tate, Cade Mack, Sofia Carozza, David Farynyk, Mehdi Bourahla, Oluwamayomikun Adeboye, Grace Cooke, Olivia Berglund, Riya Dahima, Melinda Luo, Vrushali Dhongade, George S. Usmanov, Kelly White, Amanda M. Bernal, Ross Zafonte, Shrikanth Narayanan, Minwoo Lee, Matthias R. Mehl, Min Shin

**Affiliations:** 1 Department of Neurology, Brigham and Women’s Hospital, Boston, MA 02115 USA; 2 Harvard Medical School, Boston, MA 02115 USA; 3 Network Science Institute, Northeastern University, Boston, MA 02115 USA; 4 Department of Computer Sciences, College of Computing and Informatics, University of North Carolina Charlotte, Charlotte, NC 28223 USA; 5 Department of Psychology, College of Science, University of Arizona, Tucson, AZ 85721 USA; 6 Department of Physical Medicine & Rehabilitation, Spaulding Rehabilitation Hospital, Boston, MA 02129 USA; 7 Ming Hsieh Department of Electrical and Computer Engineering, University of Southern California, Los Angeles, CA 90089 USA

**Keywords:** Biomarkers, Computational biology and bioinformatics, Health care, Neurology, Neuroscience

## Abstract

Social interaction supports brain health and recovery after neurological injury. Yet no validated tool exists for real-time measurement in individuals with and without neurological deficits. We developed SocialBit, a lightweight, privacy-preserving machine learning algorithm that detects social interactions using ambient audio features on a commercial smartwatch. In a prospective validation study, we evaluated SocialBit against livestream minute-by-minute human-coded ground truth in 153 hospitalized stroke patients who wore the device for up to 8 days, generating 88,918 min of observation. In these patients, the stroke severity and cognition spanned broad clinical ranges (NIH Stroke Scale 0–25; Montreal Cognitive Assessment 8–30), and 24 patients had aphasia across diverse subtypes, including severe presentations. SocialBit achieved high overall performance (sensitivity 0.87, specificity 0.88, area under the curve 0.94) and maintained accuracy in patients with language deficits (AUC 0.93). Despite lower temporal sampling, SocialBit produced interaction frequency distributions closely matching minute-by-minute human coding. Performance was robust across environments and interaction types. Of clinical relevance, SocialBit showed that patients with more severe strokes engaged in less social interaction, paralleling human-coded results. SocialBit is an accurate digital biomarker of social interaction with potential applications in remote monitoring and clinical trials.

**Supplementary Information:**

The online version contains supplementary material available at 10.1038/s41598-026-37746-x.

## Introduction

Engaging in social interactions is one of the most complex cognitive tasks performed by humans. It requires the close coordination of multiple cognitive processes, including meaning making, narrative coherence, perspective-taking, and non-verbal synchrony. These functions rely on distributed neural systems across frontal, temporal, and parietal cortices that were shaped by evolutionary pressures^1,2^. For instance, functional neuroimaging studies show that coordinated activity in the medial prefrontal cortex, temporoparietal junction, and fusiform gyrus contribute to processes such as empathy, trait inference, and facial recognition that are required for social interactions^3^. These areas develop together from infancy through processes of activity-dependent plasticity that are driven by the child’s experiences of the social environment^4^. Collectively, engagement in social interactions represents a high-order, multimodal expression of cognitive function.

Beyond serving as a marker of cognitive health, social interactions also play a protective role in long-term health outcomes^5^. Robust evidence shows that social connections increase longevity and reduce cardiovascular disease, cognitive decline, and depression with effects comparable to smoking or obesity^6^. The World Health Organization now lists “social support networks” as a determinant of health, alongside income, education, and access to care. When individuals are integrated into social communities, biological systems function more adaptively; conversely, persistent social isolation leads to elevated stress responses and increased allostatic load^7^. Despite the clear evidence of its importance, social interaction remains difficult to measure in clinical settings, and there are no widely implemented tools for passive, real-time social monitoring.

The implications of this measurement gap are particularly consequential for individuals recovering from stroke. Stroke survivors frequently experience social isolation due to speech, cognitive, and mobility impairments—factors that also impair their ability to complete self-report instruments^8^. This isolation occurs precisely during a time when social stimulation may offer the greatest benefit. A large body of preclinical evidence shows that enriched environments that include social interaction promote neurogenesis, synaptic remodeling, and functional recovery after stroke^9,10^. These effects are mediated by upregulation of neurotrophic factors and increased neuronal activity in peri-infarct tissue^9^. Yet, inpatient rehabilitation settings are often devoid of such social stimulation^11^. Even if social stimulation was prioritized, we lack scalable tools to track and optimize social engagement during the recovery process.

**Fig. 1:**
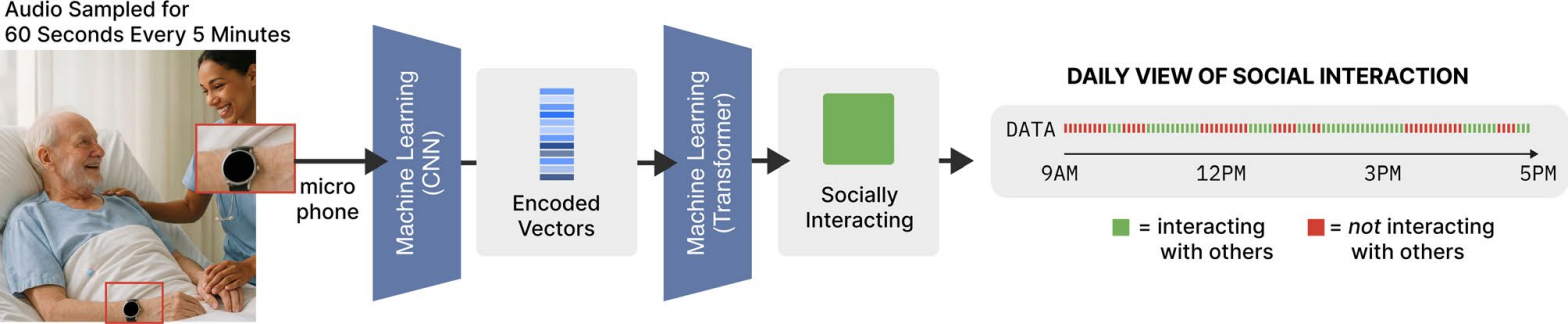
SocialBit is a Smartwatch App that Measures Social Interaction.This figure is a schematic representation, and the displayed interaction and noninteraction bars do not reflect SocialBit’s actual temporal resolution. Notably, social interaction was defined as any utterance spoken by or to the patient with another person. Image credit: The left-side image in Fig. 1 is adapted from “Compassionate healthcare nurse providing gentle care to elderly patient in modern hospital room” by Jelena, via Adobe Stock (Image ID: #1554007103).

To address this need, we developed SocialBit, a lightweight machine learning algorithm that detects social interaction using ambient audio features collected passively on commercial smartwatches (Fig. 1)^12^. SocialBit does not store raw audio or use natural language processing, preserving privacy while enabling deployment in persons with and without language difficulties. Prior methods for interaction detection have relied on sensors worn by all parties, GPS tracking, or intrusive recording devices, which were not suited for cognitively, linguistically, or motor-impaired individuals^13–15^. In contrast, SocialBit aims to be technically parsimonious and universally accessible.

In this study, we tested the accuracy of SocialBit in a cohort of 153 hospitalized stroke patients with diverse neurological abilities. Against minute-by-minute human-coded ground truth of patients’ interactions via live stream video, we evaluated the algorithm’s performance under varying conditions.

## Results

### Participants

We enrolled 153 hospitalized patients with ischemic stroke between June 2021 and March 2025. Figure S1 shows the participant flow diagram. As detailed in Table 1, participants had a mean age of 66 years (SD 13.9), and 53% were male. Stroke severity spanned a broad clinical range, with NIH Stroke Scale scores from 0 to 25 (median 2, IQR 0.00, 4.00), encompassing mild (0–5), moderate (6–15), and severe (> 15) stroke presentations. Cognitive assessments also showed a range, with Montreal Cognitive Assessment score from 8 to 30 (mean 23.6, SD 4.5). Aphasia was present in 24 participants (16%), including global (*n* = 7), Wernicke’s (*n* = 2), Broca’s (*n* = 5), mixed (*n* = 5), and unspecified subtypes (*n* = 5). This represented a heterogenous aphasia cohort mostly with moderate to severe stroke presentations. Most participants (84%) spoke English only, while 15% were bilingual or primarily Spanish-speaking.

**Table 1:** Baseline characteristics of Participants.

	Sample	Missing
	N = 153	N (%)
Sex		0 (0)
Male	81 (53)	
Female	71 (46)	
Other	1 (1)	
Age		0 (0)
Min	26	
Max	94	
Mean (SD)	65.61 ± 13.84	
Race		3 (2)
White	116 (77)	
Black	25 (17)	
Asian	3 (2)	
American Indian/ Alaska Native	1 (1)	
More than one	5 (3)	
Ethnicity		1 (1)
Not Hispanic or Latino	144 (95)	
Hispanic or Latino	8 (5)	
Language		0 (0)
English only	129 (84)	
Multiple	20 (13)	
Spanish only	2 (1)	
Other	1 (1)	
Education		6 (4)
< High school diploma	9 (6)	
High school diploma	28 (19)	
Some college or associate	42 (27)	
Bachelor’s degree	41 (28)	
Graduate degree	27 (18)	
Employment		4 (3)
Retired	71 (46)	
Paid work	65 (44)	
Unemployed	8 (5)	
Unpaid work	2 (1)	
Other	3 (2)	
Income		29 (19)
Under $25,000	19 (12)	
$25,000-$49,000	5 (3)	
$50,000 to $74,999	20 (16)	
$75,000 to $99,999	16 (13)	
$100,000 and over	53 (43)	
Modified Rankin Scale		7 (5)
Min	0	
Max	5	
Mean (SD)	2.23 ± 1.71	
NIH Stroke Scale		2 (1)
Min	0	
Max	25	
Median (IQR)	2 (0.00, 4.00)	
Montreal Cognitive Assessment		19 (12)
Min	8	
Max	30	
Mean (SD)	23.6 (4.5)	
Aphasia, n (%)		1 (1)
Total	24 (16)	
Global	7 (5)	
Wernicke’s	2 (1)	
Broca’s	5 (3)	
Mixed	5 (3)	
Unknown	5 (3)	
Delirium (Confusion Assessment Method), n (%)^ǂ^		3(2)
Present		
	4 (3)	N/A
Absent	133 (86)	
Unable to assess	10 (7)	
^ǂ^ Delirium via the Confusion Assessment Method was assessed by clinical nurse in only certain patients at risk of delirium. Unable to assess is usually due to concomitant aphasia.		

### Data collected

The ground truth dataset included 88,918 min (~ 1,482 h) of human-coded data. The SocialBit dataset included 14,045 min (~ 234 h) of algorithm-generated data. The ground truth data, of which SocialBit data are a subset, spanned 325 hospital days in total, with an average of 2.21 coded days per participant. Of the total minutes, 66,576 min (~ 1,110 h) were collected at Brigham and Women’s Hospital, and 12,109 min (~ 202 h) at Spaulding Rehabilitation Hospital. The median amount of coding per participant was 428 min (interquartile range [IQR] 525.5), with a mean of 604.88 min (SD 566.37). Coding duration per participant was higher at Spaulding (median 888 min, IQR 764) compared to Brigham and Women’s (median 378 min, IQR 497). There were no adverse events during the data collection.

**Fig. 2:**
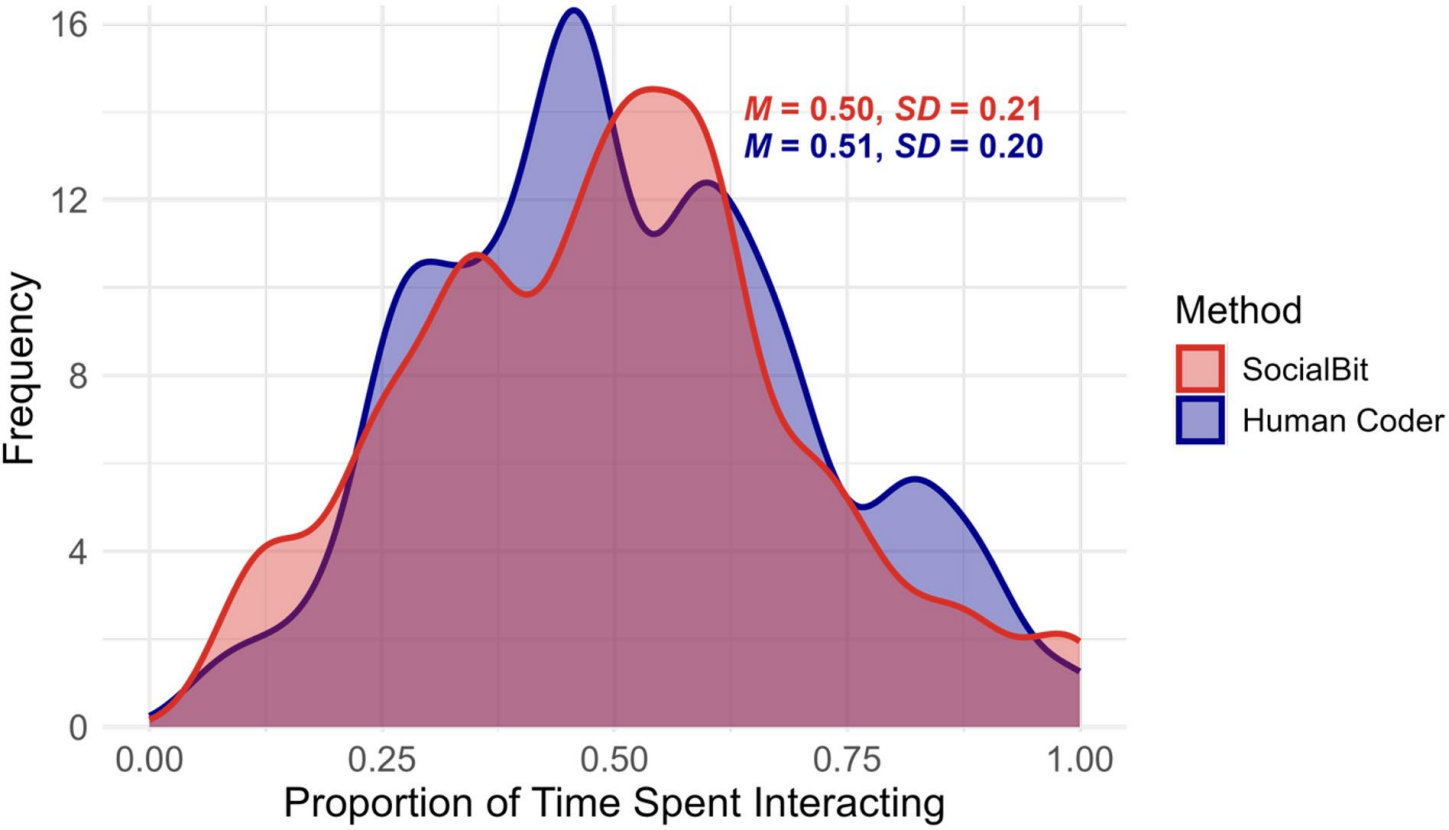
Distribution of the Proportion of Time Spent Interacting in Human-Coded Data Versus SocialBit Data.

To evaluate potential bias from SocialBit’s 1-in-5-minute sampling strategy, we compared kernel density plots of social interaction frequency with minute-by-minute human coding (Fig. 2). The proportion of time spent interacting was the number of minutes coded as social interactions divided by all minutes observed by human coders or SocialBit. The distributions overlapped closely, with comparable means and standard deviations (human coding: M = 0.51, SD = 0.20; SocialBit: M = 0.50, SD = 0.20).

### Algorithm’s overall accuracy

**Fig. 3:**
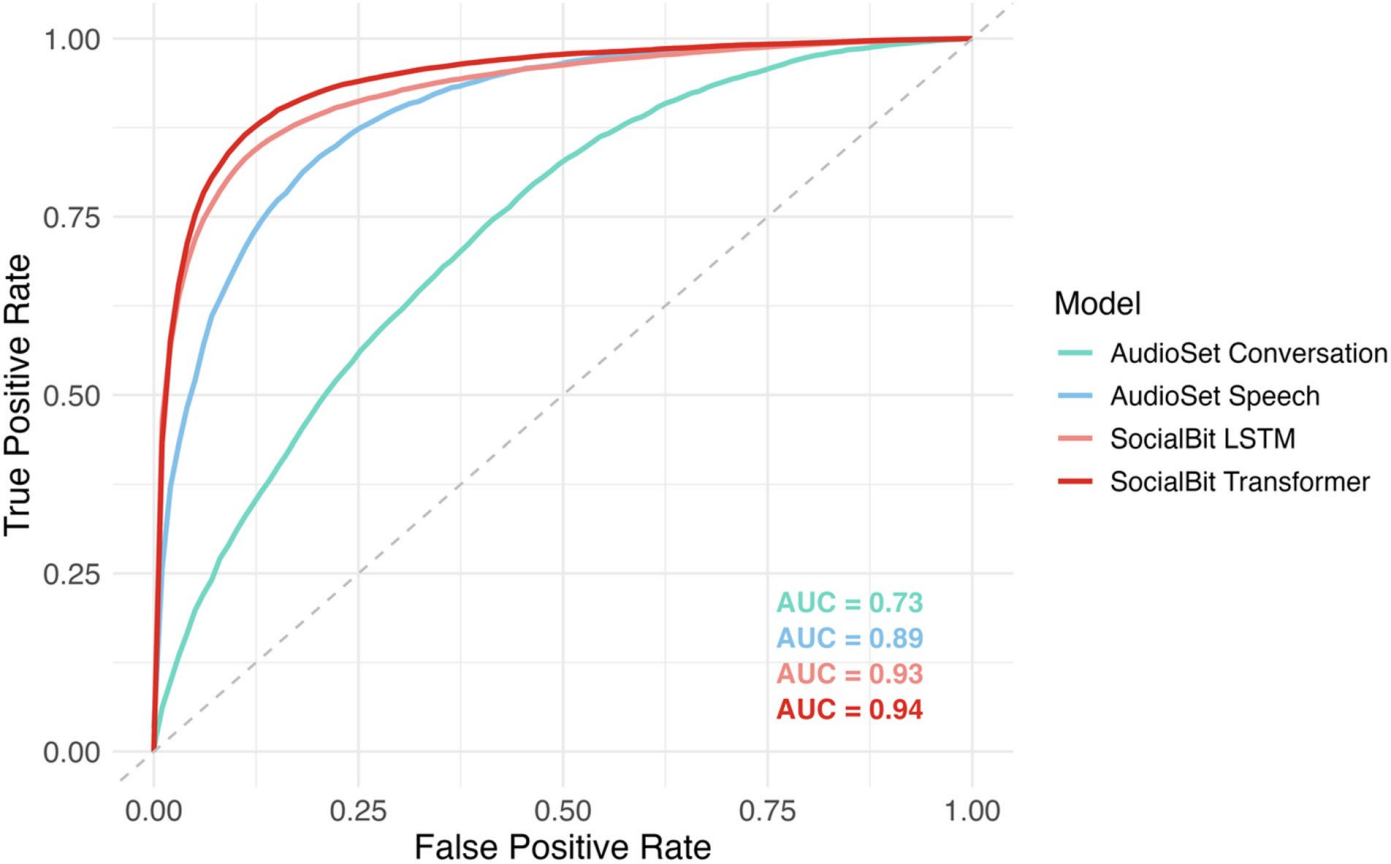
Receiver Operating Characteristic of SocialBit Algorithm Versions.

As seen in Table 2; Fig. 3, the SocialBit LSTM and SocialBit Transformer fine-tuned algorithms performed better than the benchmark AudioSet Speech and AudioSet Conversation classifiers. SocialBit LSTM and SocialBit Transformer out-performed AudioSet Speech by 4.9% and 6.1% in balanced accuracy, respectively. The SocialBit Transformer algorithm achieved the highest overall performance, with a mean balanced accuracy of 0.87 and AUC of 0.94.

**Table 2:** Overall accuracy of socialbit Algorithms.

	Sensitivity	Specificity	Balanced Accuracy	AUC
AudioSet Speech	0.83 ± 0.04	0.80 ± 0.01	0.82 ± 0.02	0.89 ± 0.02
AudioSet Conversation	0.77 ± 0.06	0.57 ± 0.09	0.67 ± 0.02	0.73 ± 0.03
SocialBit LSTM	0.84 ± 0.04	0.88 ± 0.03	0.86 ± 0.02	0.93 ± 0.02
SocialBit Transformer	0.87 ± 0.04	0.88 ± 0.04	0.87 ± 0.02	0.94 ± 0.02

To further assess subject-level generalization, we examined the distribution of test accuracy for individual patients derived from the 5-fold cross-validation. The model achieved a mean participant-level accuracy of 0.86 (SD = 0.10, *n* = 139). This variance was partially attributable to data volume; the standard deviation in accuracy dropped by half (from 0.12 to 0.06) when comparing patients with fewer than 100 samples to those with 100 or more. While variance was higher at the individual level compared to the aggregate fold level, the mean performance remained consistent with the overall model metrics.

For specificity, the SocialBit Transformer outperformed the AudioSet Speech and AudioSet Conversation classifier by 8.0% and 31.6%, respectively, reducing false positives from non-social sounds (e.g., background TV). For sensitivity, SocialBit Transformer exceeded AudioSet Speech and AudioSet Conversation by 4.8% and 13.0%, respectively, demonstrating strong detection even in noisy or ambiguous interaction settings. We report subsequent analyses using the SocialBit Transformer, which showed higher performance across sensitivity, specificity, balanced accuracy, and AUC.

### Algorithm’s accuracy in patients with aphasia

**Fig. 4:**
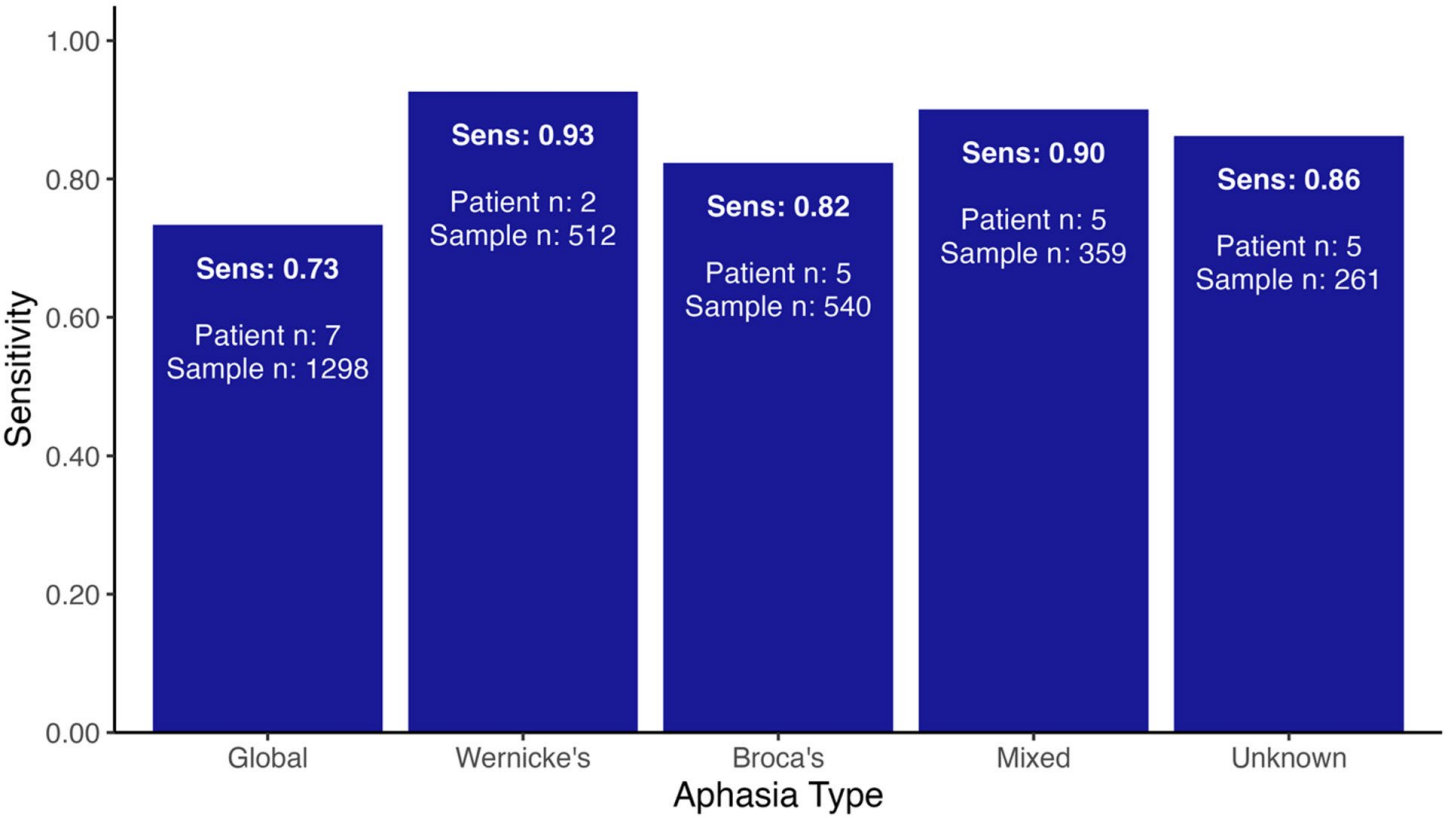
SocialBit sensitivity by aphasia subtype.

SocialBit maintained high performance in patients with aphasia (*n* = 24), achieving 0.82 sensitivity, 0.87 specificity, 0.84 balanced accuracy, and 0.93 area under the curve (AUC) (Table 3). Compared to patients without aphasia, this represented a moderate decrease of 6.8% in sensitivity, 2.2% in specificity, 4.5% in balanced accuracy, and 1.1% in AUC. Figure 4 displays SocialBit sensitivity across aphasia subtypes, highlighting variability by subtype with the lowest sensitivity observed in global aphasia. The reduction in sensitivity across all patients with aphasia may be because of fewer speech contributions from the patient with aphasia, and social interactions in such patients being briefer.

**Table 3:** SocialBit accuracy in patients with Aphasia^§^.

	Sensitivity	Specificity	Balanced Accuracy	AUC
Patients with Aphasia	0.82 ± 0.04	0.87 ± 0.10	0.84 ± 0.04	0.93 ± 0.03
Patients without Aphasia	0.88 ± 0.05	0.89 ± 0.01	0.88 ± 0.02	0.94 ± 0.02

^§^These results are based on the best-performing version, SocialBit Transformer.

### Algorithm’s clinical validity

**Fig. 5:**
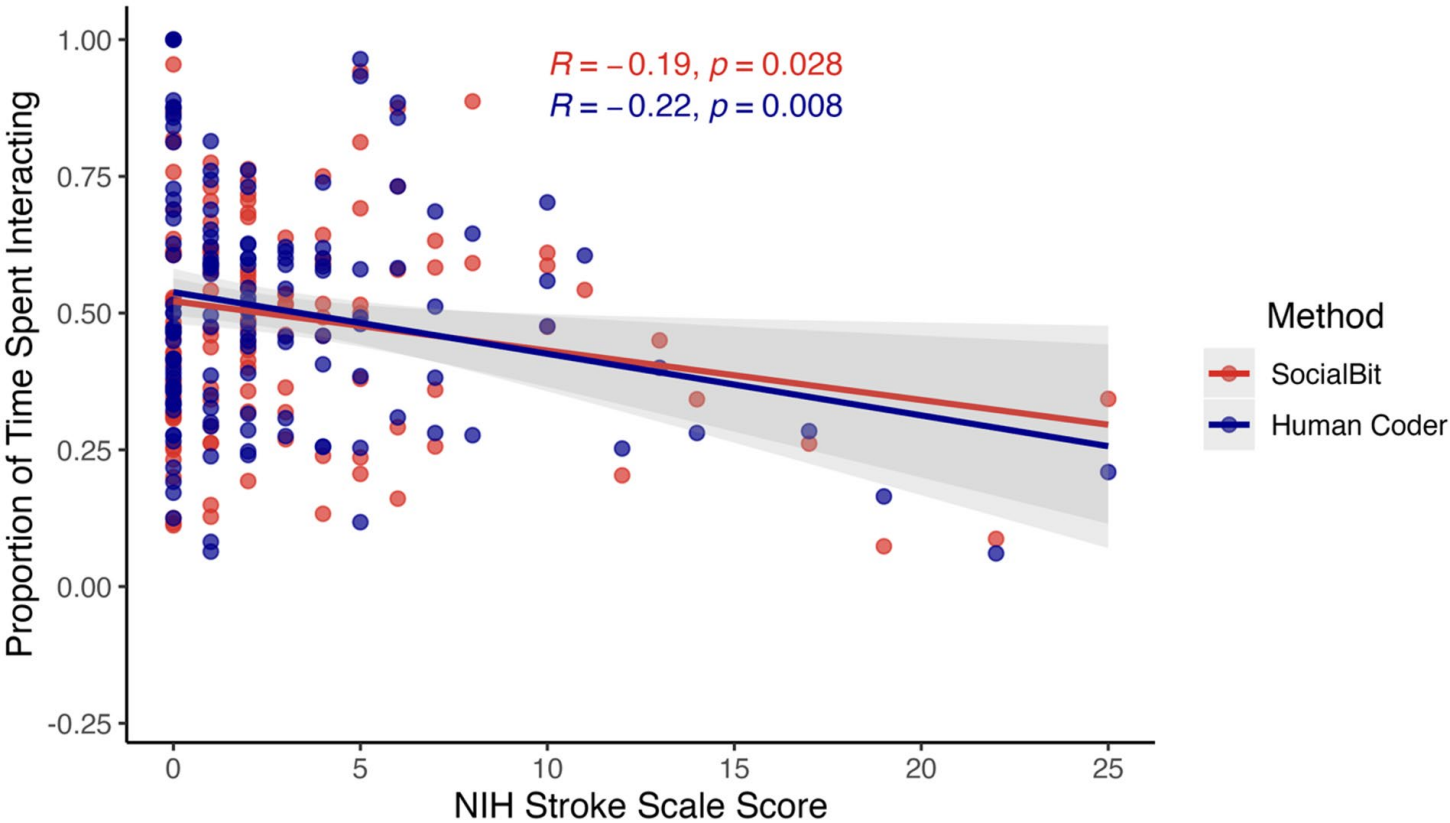
SocialBit and Ground-truth (i.e., Human Coder) Effect Estimates for the Association between Social Interaction Frequency and Stroke Severity.

We evaluated the clinical validity of SocialBit by examining its relationship with bedside-assessed stroke severity and comparing it to ground-truth human-coded interaction data. Higher National Institutes of Health Stroke Scale (NIHSS) scores (M = 3.0, SD = 4.4; range = 0–25) correlated significantly with fewer social interactions measured by SocialBit (*r* = − 0.19, *p* = 0.029) and by human coders (*r* = − 0.22, *p* = 0.008). Specifically, each 1-point increase in NIHSS corresponded to a 0.9% decrease in SocialBit-estimated interaction time and a 1.1% decrease in ground-truth-coded interaction time. These findings demonstrate that SocialBit captures meaningful social effects linked to stroke severity with high psychometric fidelity (Fig. 5).

### Algorithm performance across interaction characteristics

We evaluated the sensitivity of SocialBit across six features of social interaction: depth, tone, number of speakers, interaction partner, communication modality, and spoken language (Table 4). Because annotations were available only for positive samples, we report sensitivity only.

**Table 4:** SocialBit sensitivity across varying dimensions of social Interactions^§^.

Interaction Category	Sensitivity
Depth 1 (325) 2 (1,393) 3 (3,642) 4 (1,001) 5 (69)	0.55 ± 0.06 0.79 ± 0.05 0.91 ± 0.04 0.96 ± 0.05 0.96 ± 0.04
Tone −2 (19) −1 (137) 0 (4,925) +1 (1,295) +2 (94)	0.99 ± 0.02 0.95 ± 0.08 0.87 ± 0.03 0.89 ± 0.07 0.86 ± 0.09
Number of Speakers 1 (4,757) 2 (1,457) 3+ (309)	0.86 ± 0.04 0.90 ± 0.05 0.93 ± 0.07
Interaction Partners Medical Personnel (3,218) Family/Friends (1,680) Caregiver (1,618) Other Patient (205) Stranger (646) Can’t Tell (441)	0.88 ± 0.02 0.88 ± 0.07 0.86 ± 0.07 0.82 ± 0.11 0.92 ± 0.01 0.90 ± 0.02
Spoken Language^ǂ^ English (6,334) Non-English (210)	0.88 ± 0.04 0.89 ± 0.14
Communication Modalities In-Person (5,566) Phone Call (907) Video Call (61)	0.87 ± 0.05 0.91 ± 0.03 0.88 ± 0.13

§ These results are based on the best-performing version, SocialBit Transformer.

ǂ Algorithm performance varied widely across participants.

#### Depth of interaction

Sensitivity increased with conversational depth: 0.55 for level 1, 0.79 for level 2, and ≥ 0.91 for levels 3–5. Shallow interactions (e.g., greetings) were harder to detect, while deeper exchanges produced sustained features aiding detection.

#### Tone

Sensitivity was high across tones, with slightly better performance in negatively valanced interactions. Neutral (76%) and mildly positive (20%) tones (tone + 1) had sensitivities of 0.87 and 0.89; strongly positive (tone + 2) was 0.86. Negative tones yielded 0.95 (tone − 1) and 0.99 (tone − 2).

#### Number of speakers

Consistent with the idea that more speech facilitates social interaction detection, performance improved with more speakers: 0.86 for one-on-one, 0.90 for two speakers, and 0.93 for three or more.

#### Interaction partner

Sensitivity was consistent across partners: 0.88 for clinicians/family, 0.82 for patients, 0.86 for caregivers, 0.92 for strangers, and 0.90 for unidentified.

#### Spoken Language

Performance was similar for English (0.88) and non-English (0.89; *n* = 210 samples) interactions.

#### Communication modality

Sensitivity varied across communication formats. Sensitivity was 0.87 for in-person, 0.91 for phone, and 0.88 for video calls, with highest detection on phone calls.

### Algorithm performance across acoustic and environmental conditions

We tested SocialBit robustness in four real-world conditions that introduce acoustic complexity: (1) side conversations, (2) television sounds, (3) care setting, and (4) smartwatch hardware (Table 5.).

**Table 5.** SocialBit Performance Across Environmental and Sensor Conditions^§^

	Sensitivity	Specificity	Balanced Accuracy	AUC
Side Conversation				
Present (4,793)	0.86 ± 0.04	0.82 ± 0.03	0.84 ± 0.02	0.92 ± 0.01
Absent (9,252)	0.87 ± 0.05	0.92 ± 0.04	0.90 ± 0.03	0.95 ± 0.02
Television Sound				
Present (4,907)	0.82 ± 0.06	0.89 ± 0.04	0.85 ± 0.02	0.92 ± 0.02
Absent (9,138)	0.89 ± 0.04	0.87 ± 0.03	0.88 ± 0.02	0.95 ± 0.01
Care Setting				
Hospital (12,227)	0.86 ± 0.04	0.88 ± 0.03	0.87 ± 0.02	0.94 ± 0.01
Rehabilitation (1,818)	0.92 ± 0.05	0.88 ± 0.12	0.90 ± 0.05	0.96 ± 0.02
Smartwatch Hardware				
TicWatch Pro 3 (8,448)	0.87 ± 0.07	0.89 ± 0.06	0.88 ± 0.03	0.94 ± 0.02
Galaxy Watch5 Pro (5,597)	0.87 ± 0.02	0.87 ± 0.01	0.87 ± 0.01	0.94 ± 0.01

^§^These results are based on the best-performing version, SocialBit Transformer.

#### Side conversation

There was stable performance in the presence of side conversations. The model had a balanced accuracy of 0.84 and an AUC of 0.92 when side conversations were present, compared to 0.90 and 0.95 when side conversations were absent.

#### Television sound

Television presence had a minor effect on performance, primarily reducing sensitivity. The model had a balanced accuracy of 0.85 and AUC 0.92 with television present, compared to 0.88 and 0.95 with television absent.

#### Care setting

Performance was consistent across care settings: In the hospital, the model had a balanced accuracy of 0.87 and an AUC of 0.94. In the rehabilitation setting, the model had a balanced accuracy of 0.90 and an AUC of 0.96.

#### Smartwatch hardware

Performance remained equivalent across different smartwatch devices. For the TicWatch Pro 3, the model had a balanced accuracy of 0.88 and AUC of 0.94. For the Galaxy Watch5 Pro, the model had a balanced accuracy of 0.87 and AUC of 0.94.

## Discussion

Our findings provide evidence for SocialBit as an accurate, universally accessible, and privacy-preserving sensor for detecting social interactions in patients with diverse neurological abilities. In 153 stroke survivors, SocialBit achieved high sensitivity (0.87), specificity (0.88), and overall discriminative ability (AUC 0.94) when benchmarked against minute-by-minute human coding and when benchmarked against existing speech and conversation classifiers. SocialBit maintained high accuracy even in patients with aphasia, a population historically excluded from survey-based research. The algorithm captured meaningful variation across key features of social interaction and performed reliably across environmental conditions and smartwatch devices.

Our findings build on recent advances in ambient audio sensing for social behavior. Prior methods have shown the feasibility of detecting face-to-face interactions using smartphone audio, foreground speech localization, and multi-party wearables^13–17^. For example, Liang et al. introduced a smartwatch model detecting conversational turn-taking via foreground speech and speaker change detection^16^. SocialBit complements these efforts by expanding detection to interactions without clear speaker alternation or linguistic symmetry—common in patients with aphasia, cognitive impairment, or serious illness. By using a single wearable without requiring turn-taking or speech content, SocialBit offers a technically simple, socially inclusive framework for real-world interaction monitoring.

Our findings can be contextualized relative to prior observational studies of inpatient stroke social activity, particularly the work by Bernhardt et al.^11^ In that multicenter study of 64 patients within 14 days of stroke, observed between 8 am and 5 pm, patients were with others for ~ 40% of the day. Our study shared key design features, including daytime observation in hospitalized stroke populations and documentation of the presence of other people. However, we observed higher rates of social interaction, approaching 50% by both human coding and SocialBit. Several factors may explain this difference. Our observations used higher temporal resolution, with one-minute human coding and five-minute SocialBit estimates, which may capture brief or fleeting interactions missed by ten-minute sampling. Our cohort also included both acute stroke units and inpatient rehabilitation settings, where social exposure may differ. Finally, social interaction after stroke likely varies across hospital environments and care models. Together, these findings suggest that observed social interaction after stroke varies by setting and measurement approach, warranting further study.

Our findings align with a growing field that uses voice as a digital biomarker for health. Voice has been linked to physical and mental well-being, reflecting changes in respiratory function, emotional state, and neurological status through passive monitoring^18^. This interest has expanded into cognitive health, where patterns in spontaneous speech—such as lexical diversity, syntactic complexity, prosody, pause duration, and turn-taking—are associated with mild cognitive impairment, early dementia, and functional decline^19^. Disruptions in these features often signal altered neurocognitive status, including slower responses, reduced topic maintenance, and diminished conversational synchrony^20^. However, most approaches depend on natural language processing and cooperative verbal output, limiting their use in everyday naturalistic settings. SocialBit complements this work by shifting focus from speech content to non-lexical acoustic features.

SocialBit’s passive detection of social interactions has practical applications across medical conditions. In clinical research, SocialBit can serve as an objective, longitudinal outcome for clinical trials, capturing aspects of quality of life that are central to recovery yet invisible to traditional clinical metrics. Studies have shown that relationships are central to quality of life, with strong social ties linked to greater resilience, emotional well-being, and functional independence^5^. In stroke recovery, social engagement is a marker and modifiable target of cognitive rehabilitation^21,22^. By enabling continuous measurement without reliance on self-report, SocialBit is particularly well suited for patients with aphasia or cognitive impairment, who are often excluded from existing assessments.

In clinical workflows, SocialBit could support remote monitoring by identifying early reductions in social engagement that may precede cognitive or functional decline, enabling timely intervention^23,24^. In inpatient and rehabilitation settings, social interaction metrics could complement physical and cognitive measures to track recovery trajectories and tailor therapy intensity or social enrichment strategies. Over longer time horizons, SocialBit may enable outcome assessment beyond discharge, supporting personalized rehabilitation, caregiver engagement, and evaluation of interventions aimed at reducing isolation. By quantifying real-world sociality, SocialBit provides a foundation for operationalizing social prescriptions and establishing social interaction as a measurable vital sign within routing clinical care.

This study has limitations. First, although conducted in naturalistic hospital settings with continuous minute-level labeling, the algorithm may need further tuning in home or community contexts. Future studies should explore longer-term use in ambulatory populations. Second, although our cohort was clinically and demographically diverse, most interactions were in English. Third, the study of patients with severe stroke presentations and aphasia were limited, representing an important area for future work. Fourth, the current model classifies the presence of interaction but not its depth, tone, or quality, which are features important to functional outcomes. Finally, integrating social sensing into clinical workflows will require shifts in culture, reimbursement, and regulatory frameworks to support behavioral vital signs as routine metrics.

By passively detecting real-world interactions in individuals with diverse neurological abilities, SocialBit fills a gap in digital health. It provides a technically parsimonious, privacy-preserving, and inclusive method for measuring a core dimension of brain health. Looking ahead, SocialBit could help establish social interaction as a routinely monitored dimension of clinical care, serving as both a research endpoint and a practical tool for guiding rehabilitation and preventing isolation in clinical populations.

## Methods

### Study design and participants

We provide a full description of the study rationale, design, and procedures in a protocol paper^12^. In brief, we conducted a prospective observational study to test the validity of SocialBit. Our primary aim was to assess the accuracy of SocialBit in detecting minute-level social interactions, using human observation as the ground truth.

We recruited participants in consecutive series while they were inpatients at Brigham and Women’s Hospital from June, 2021 to March, 2025. Inclusion criteria were: (1) Diagnosed with an acute ischemic stroke defined clinically with support from imaging and (2) 18 years old or older. Exclusion criteria were: (1) On Comfort Measures Only (a patient end- of- life care plan that focuses on pain relief and quality of life), (2) Diagnosed with dementia prior to stroke in the medical record, (3) Unable to obtain informed consent from the patient or patient decision maker, and (4) Patient or patient decision maker is unable to understand or speak English well enough to complete surveys.

### Algorithm development

We developed the SocialBit algorithm to detect minute-level social interaction using ambient audio captured from a consumer-grade smartwatch. Our primary goal was to balance high model performance with technical feasibility for clinical research, including battery life constraints and privacy protections.

For algorithm development, we began with YAMNet^25^, a pre-trained deep neural network designed for general-purpose audio classification using the AudioSet ontology of 521 sound events^26^. YAMNet is based on the MobileNet v1 architecture^27^, containing 3.7 million parameters and capable of running in real time on a smartwatch while preserving battery life. It processes non-overlapping 0.96-second windows of raw audio and outputs class probability scores. For our purposes, we extracted the 1024-dimensional penultimate layer embeddings, which provide rich, compressed feature representations that exclude sensitive content such as specific words or speaker identity. Notably, in preliminary analyses, we evaluated commonly used voice activity detection approaches, including WebRTC VAD and Silero VAD, as potential baselines. However, these methods, which are optimized for binary speech detection in telephony settings, showed limited robustness in our on-body, real-world recordings with overlapping speakers and environmental noise; we therefore selected YAMNet Speech and Conversation classes as more task-relevant and rigorous baselines for social interaction detection. The algorithm needed no preprocessing or artifact mitigations strategies.

We used YAMNet embeddings as input to fine tune sequential models that could learn patterns of social interaction over time. Specifically, we trained two models: a Long Short-Term Memory (LSTM) network^28^ and a Transformer model^29^. Both models were designed to classify each one-minute segment of audio as containing social interaction or not. The LSTM model captured temporal dependencies by processing the sequence of YAMNet embeddings step-by-step, while the Transformer model used parallelized self-attention to evaluate all time steps simultaneously, offering computational advantages and improved handling of longer-range dependencies.

The SocialBit LSTM model architecture comprised a bidirectional LSTM layer with 350 units, followed by a dropout layer (75%), and a dense two-unit softmax output layer. The SocialBit Transformer model architecture included two sequential Transformer units, each composed of layer normalization, multi-head attention (six heads, 768 dimensions), dropout (35%), residual connections, and an additional normalization step. These were followed by a 1D convolutional layer (nine filters, kernel size 1 × 1), a fully connected layer with 35 units, and a final dropout layer. Notably, to preserve battery life, the SocialBit algorithms collected audio features for 1 out of every 5 min. In practice, the realized sampling yield was slightly lower than the nominal 20% due to technical factors, including intermittent time synchronization issues and transient software or data upload failures. These issues occasionally prevented scheduled sampling windows from being recorded but did not reflect systematic resets or errors in the sampling logic. During the study, we deployed system stability updates to maintain compatibility with evolving Wear OS versions and to improve data reliability.

For transparency and reproducibility, we provide layer-by-layer specifications of the SocialBit LSTM and Transformer architectures as Figure S2. This includes model structure, hyperparameters, and implementation details in TensorFlow/Keras.

For this study, we performed YAMNet feature extraction on-device on the smartwatch and conducted backend model training and evaluation off-device. Fully on-device inference represents a future implementation goal and was not assessed in this study.

### Deployment in clinical setting

Participants wore a smartwatch running the SocialBit algorithm each day from 9:00 am to 5:00 pm for up to 5 days at Brigham and Women’s Hospital, or up to 3 days at Spaulding Rehabilitation Hospital. During this period, trained human coders used a HIPAA-compliant livestream system to label the presence or absence of social interaction and interaction characteristics minute-by-minute in a REDCap ground truth table (Table S1). Social interaction was defined as any utterance spoken by or to the patient with another person. Utterances included fragmented speech, non-sensical speech, or non-verbal sounds as may occur in patients with aphasia (for detailed guidelines on making coding judgements, see protocol)^12^. We also administered cognitive testing and standardized questionnaires to assess social connectedness, mood, and function at enrollment and again at 3 months. We determined aphasia status and subtype by detailed review of the medical record, including neurology and speech-language pathology documentation. We classified aphasia as global, Broca’s, Wernicke’s, mixed, or unspecified based on documented expressive and receptive language deficits. All participants provided written informed consent. The Mass General Brigham Institutional Review Board approved all study procedures. All methods were performed in accordance with the relevant guidelines and regulations.

### Evaluation of socialbit performance

We evaluated two fine-tuned SocialBit algorithms, SocialBit LSTM and SocialBit Transformer, against the two most corresponding Google AudioSet classifiers as benchmarks, AudioSet Speech and AudioSet Conversation. We evaluated the raw AudioSet model’s output on these classes as a baseline, using the maximum score across each 1-minute window. Any time-points missing human-adjudicated data (such as when patients were being cleaned or changed) were removed. As described in our protocol, we determined sample size by iterative performance metrics and standards for deep learning algorithms in prior studies^12^.

We evaluated the performance of the models using standard classification metrics: sensitivity, specificity, balanced accuracy, and area under the receiver operating characteristic curve (AUC). Sensitivity was the proportion of correctly identified positive samples, while specificity was the proportion of correctly identified negative samples. Balanced accuracy was the calculated average of sensitivity and specificity. AUC was the trade-off between sensitivity and specificity across thresholds.

We implemented participant-level 5-fold cross-validation to ensure that data from any individual participant appeared in only one-fold, preventing overlap between training and testing sets. For the AudioSet Speech and AudioSet Conversation baseline models, within each training fold, we selected classification thresholds (τ) that maximized the harmonic mean of sensitivity and specificity.

For the fine-tuned SocialBit LSTM and SocialBit Transformer models, each training set (comprising four folds) was further split into training and validation subsets in a 3:1 ratio. We used validation subsets for hyperparameter tuning, including early stopping. This procedure yielded 20 trained models per architecture across the five outer folds. To reduce training bias from class imbalance, we applied under-sampling of the majority class randomly at the sample level during model training^30^.

We performed hyperparameter optimization using Bayesian optimization with validation accuracy as the objective, tuning hyperparameters independently within each training fold. Across folds, we observed highly consistent selected hyperparameter configurations and validation performance for both the LSTM and Transformer models; therefore, we adopted a single representative configuration per architecture for all reported analyses. The optimized hyperparameters, search ranges, and selected values are reported in Table S2.

For clinical validation, we tested whether SocialBit models produced effect estimates comparable to the human-coded ground truth. We evaluated the face-valid hypothesis that higher levels of stroke severity are related to lower social interaction frequency (indicating the socially isolating effects of stroke). We correlated stroke severity (NIH Stroke Scale) with social interaction frequency, defined as the proportion of observed minutes with interaction based on human coding and SocialBit. Effect estimates that are comparable in magnitude provide psychometric evidence that SocialBit can be used as a research algorithm on par with ground-truth human codings.

Finally, we conducted secondary analyses to assess SocialBit’s robustness across diverse conditions. First, we examined sensitivity by interaction characteristics—depth, tone, number of speakers, partner type, modality, and language—using human-coded annotations. Second, we evaluated balanced accuracy and AUC across challenging environments, including side conversations, background TV, care settings, and smartwatch models. These tests assessed the algorithm’s generalizability to real-world variability.

We used R version 4.4.1 for all statistical analyses. We used the STARD (Standards for Reporting Diagnostic accuracy studies) Statement Guideline in reporting results (Table S3).

## Supplementary Information

Below is the link to the electronic supplementary material.

Supplementary Material 1

## Data Availability

The data that support the findings of this study are available from Brigham and Women’s Hospital, but restrictions apply to their availability because they were used under HIPAA regulations for the current study and are therefore not publicly available. Data are, however, available from the authors upon reasonable request and with permission of Brigham and Women’s Hospital.
