# Supplementary material for: Validation of SocialBit as a smartwatch algorithm for social interaction detection in a clinical population
Source: Sci Rep. 2026 Feb 4;16:4529. doi: 10.1038/s41598-026-37746-x (PMC12873288; doi:10.1038/s41598-026-37746-x)
Supplement: Supplementary file 1 — Supplementary Material 1 [file 41598_2026_37746_MOESM1_ESM.docx]

**Supplementary Appendix**

Validation of SocialBit as a Smartwatch Algorithm for Social Interaction Detection in a Clinical Population

Dhand et al.

**Table of Contents**

## Figure S1. Participant Flow Diagram……………………………………………………………..3

## Figure S2. Keras implementation of the LSTM-based models……………………………………4

## Table S1. Ground Truth Table………………………………………………….………………….5

## Table S2. Hyperparameter search ranges and selected values…………………………………….6

## Table S2. STARD Checklist……………………………………………………………………..…………..………..7

## **Figure S1. Participant Flow Diagram**


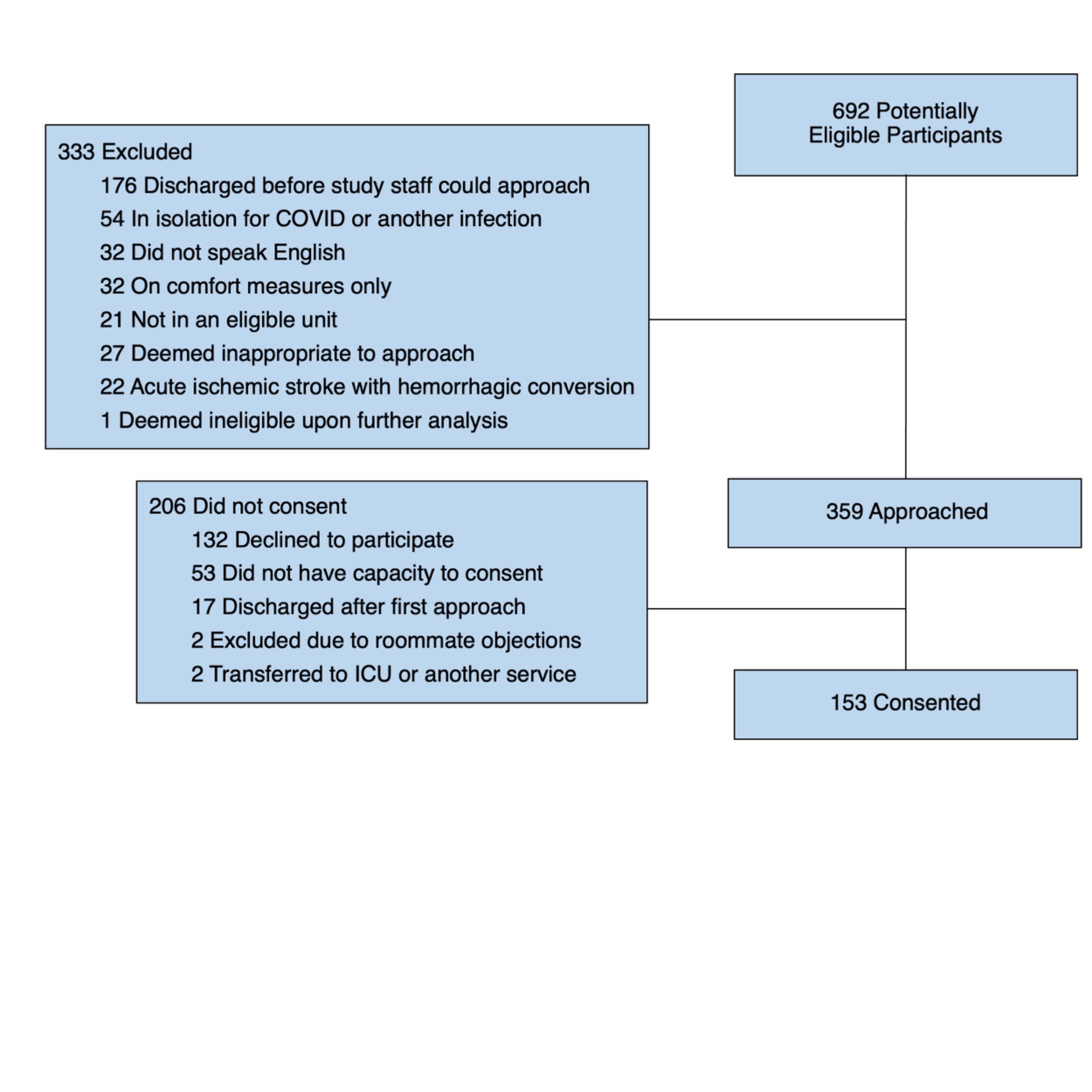


###

## **Figure S2. Keras implementation of the LSTM-based models**

| from keras import Model  from keras.layers import Input, Dense, Dropout, LayerNormalization, MultiHeadAttention, Conv1D, GlobalAvgPool1D  def build_transformer_model(feature_len=1024, num_transformers, head_size, num_heads, ff_dim, mlp_units, dropout):      inputs = Input(shape=(None, feature_len))      x = inputs      for _ in range(num_transformers):          x_in = x          x = LayerNormalization(epsilon=1e-6)(x_in)          x = MultiHeadAttention(              key_dim=head_size,              num_heads=num_heads,              dropout=dropout          )(x, x)          x = Dropout(dropout)(x)          x = x + x_in          x_ff_in = x          x = LayerNormalization(epsilon=1e-6)(x_ff_in)          x = Conv1D(filters=ff_dim, kernel_size=1, activation='relu')(x)          x = Dropout(dropout)(x)          x = Conv1D(filters=feature_len, kernel_size=1)(x)          x = x + x_ff_in      x = GlobalAvgPool1D(data_format='channels_last')(x)      x = Dense(mlp_units, activation='relu')(x)      x = Dropout(dropout)(x)      outputs = Dense(2, activation='softmax')(x)      model = Model(inputs, outputs)      return model |
| --- |

Listing 1. Keras implementation of the transformer-based model.

| from keras.layers import Input, Dense, Dropout, Bidirectional, LSTM  from keras.models import Sequential  def build_lstm_model(feature_len=1024, is_bidirectional, hidden_units, dropout):      model = Sequential()      if is_bidirectional:          model.add(Bidirectional(LSTM(hidden_units), input_shape=(None, feature_len)))      else:          model.add(LSTM(hidden_units, input_shape=(None, feature_len)))        model.add(Dropout(dropout))      model.add(Dense(2, activation='softmax'))      return model |
| --- |

Listing 2. Keras implementation of the LSTM-based model.

## **Table S1. Ground Truth Table**

| **Ground Truth Question (per minute)** | **Answer (per minute)** |
| --- | --- |
| Was the patient talking to another person? | o Yes  o No |
| Who was the patient talking with? | o Medical personnel  o Caregiver  o Other family and friends, adult or child  o Patient in other bed  o Stranger/Other  o Can’t Tell |
| How was the patient talking with the person(s)? | o In-person  o Phone call  o Video conference/chat |
| How many people was the patient talking with? (i.e. actively in conversation) | o 1  o 2  o 3 or more |
| Does the conversation contain foreign language? | o Yes  o No |
| What was the tone of the interaction?  (-2=Negative, 0=Neutral, 2=Positive) | o -2  o -1  o 0  o 1  o 2  o Can’t tell |
| What was the depth of the conversation?  (1=Superficial to 5=Deep) | o 1  o 2  o 3  o 4  o 5  o Can’t tell |
| Was the TV on?  (TV includes computer or other devices playing entertainment in a way that is audible in the room.) | o Yes  o No |
| Was there a conversation happening that the patient was not part of? | o Yes  o No |
| Was the patient completely silent during this minute?  (Did not contribute any words or sounds) | o Yes  o No |
| Anything else that was noteworthy? | __________ |

## **Table S2. Hyperparameter search ranges and selected values**

Below are the ranges and selected values for the hyperparameters. For non-categorical instances, a step size is indicated, which sometimes coarsens the parameter space.

**Transformer hyperparameters**

| **Hyperparameter** | **Selected value** | **Search range** | **Step / Categorical** |
| --- | --- | --- | --- |
| Number of transformer modules | 2 | 1 – 3 | step 1 |
| Attention head size | 768 | 16 – 1024 | step 8 |
| Number of attention heads | 6 | 1 – 24 | step 1 |
| Convolution output filters | 9 | 2 – 16 | step 1 |
| Fully connected units | 32 | 16 – 256 | step 8 |
| Dropout rate | 0.35 | 0 – 0.99 | step 0.05 |
| Learning rate | 1e-6 | {1e-2, …, 1e-6} | categorical |

**LSTM hyperparameters**

| **Hyperparameter** | **Selected value** | **Search range** | **Step / Categorical** |
| --- | --- | --- | --- |
| Bidirectional | Yes | {No, Yes} | categorical |
| Hidden units | 350 | 16 – 1024 | step 4 |
| Dropout rate | 0.75 | 0 – 0.99 | step 0.05 |
| Learning rate | 1e-5 | {1e-2, …, 1e-6} | categorical |

## **Table S3. STARD Checklist**

|  | **Section & Topic** | **No** | **Item** | **Reported on page #** |
| --- | --- | --- | --- | --- |
|  |  |  |  |  |
|  | TITLE OR ABSTRACT |  |  |  |
|  |  | 1 | Identification as a study of diagnostic accuracy using at least one measure of accuracy  (such as sensitivity, specificity, predictive values, or AUC) | 1 |
|  | ABSTRACT |  |  |  |
|  |  | 2 | Structured summary of study design, methods, results, and conclusions  (for specific guidance, see STARD for Abstracts) | 2 |
|  | INTRODUCTION |  |  |  |
|  |  | 3 | Scientific and clinical background, including the intended use and clinical role of the index test | 3 |
|  |  | 4 | Study objectives and hypotheses | 4 |
|  | METHODS |  |  |  |
|  | *Study design* | 5 | Whether data collection was planned before the index test and reference standard  were performed (prospective study) or after (retrospective study) | 13 |
|  | *Participants* | 6 | Eligibility criteria | 13 |
|  |  | 7 | On what basis potentially eligible participants were identified  (such as symptoms, results from previous tests, inclusion in registry) | 13 |
|  |  | 8 | Where and when potentially eligible participants were identified (setting, location and dates) | 13 |
|  |  | 9 | Whether participants formed a consecutive, random or convenience series | 13 |
|  | *Test methods* | 10a | Index test, in sufficient detail to allow replication | 14 |
|  |  | 10b | Reference standard, in sufficient detail to allow replication | 14-15 |
|  |  | 11 | Rationale for choosing the reference standard (if alternatives exist) | NA |
|  |  | 12a | Definition of and rationale for test positivity cut-offs or result categories of the index test, distinguishing pre-specified from exploratory | 15 |
|  |  | 12b | Definition of and rationale for test positivity cut-offs or result categories of the reference standard, distinguishing pre-specified from exploratory | 15-16 |
|  |  | 13a | Whether clinical information and reference standard results were available to the performers/readers of the index test | 15-16 |
|  |  | 13b | Whether clinical information and index test results were available  to the assessors of the reference standard | 15-16 |
|  | *Analysis* | 14 | Methods for estimating or comparing measures of diagnostic accuracy | 15-16 |
|  |  | 15 | How indeterminate index test or reference standard results were handled | 15 |
|  |  | 16 | How missing data on the index test and reference standard were handled | 15 |
|  |  | 17 | Any analyses of variability in diagnostic accuracy, distinguishing pre-specified from exploratory | 16-17 |
|  |  | 18 | Intended sample size and how it was determined | 15 |
|  | RESULTS |  |  |  |
|  | *Participants* | 19 | Flow of participants, using a diagram | 5, Figure S1 |
|  |  | 20 | Baseline demographic and clinical characteristics of participants | 5, Table 1 |
|  |  | 21a | Distribution of severity of disease in those with the target condition | 5, Table 1 |
|  |  | 21b | Distribution of alternative diagnoses in those without the target condition | NA |
|  |  | 22 | Time interval and any clinical interventions between index test and reference standard | 5 |
|  | *Test results* | 23 | Cross tabulation of the index test results (or their distribution)  by the results of the reference standard | Tables 2-5 |
|  |  | 24 | Estimates of diagnostic accuracy and their precision (such as 95% confidence intervals) | 5-6, Table 2, Figure 2 |
|  |  | 25 | Any adverse events from performing the index test or the reference standard | 5 |
|  | DISCUSSION |  |  |  |
|  |  | 26 | Study limitations, including sources of potential bias, statistical uncertainty, and generalisability | 11-12 |
|  |  | 27 | Implications for practice, including the intended use and clinical role of the index test | 11 |
|  | OTHER INFORMATION |  |  |  |
|  |  | 28 | Registration number and name of registry | NA |
|  |  | 29 | Where the full study protocol can be accessed | 13 |
|  |  | 30 | Sources of funding and other support; role of funders | 17, 19 |
|  |  |  |  |  |
